# Anisotropic Extrudate Swell from a Slit Die: A Velocity-Centre Hypothesis and Numerical Verification

**DOI:** 10.3390/polym18050652

**Published:** 2026-03-07

**Authors:** Guangdong Zhang, Xinyu Hao, Linzhen Zhou

**Affiliations:** School of Mechanical Engineering, Yancheng Institute of Technology, Yancheng 224051, China

**Keywords:** extrudate swell, mathematical modelling, slit die, viscoelastic flow, anisotropic swelling

## Abstract

While anisotropic extrudate swell in polymer processing is fundamentally driven by physical viscoelastic recovery, this paper proposes a theoretical framework to explicitly isolate and map the purely geometric and kinematic components of this phenomenon. Serving as a mathematical proof-of-concept, a multi-velocity-centre hypothesis is proposed. By introducing a semi-empirical, lumped material-flow calibration parameter, the macroscopic diameter swell ratio is mathematically extended to the discrete local flow field of a rectangular slit die. To evaluate its validity, the analytical framework is subjected to a numerical test for kinematic consistency utilizing isothermal, inelastic power-law fluid CFD simulations, thereby separating geometric mapping from complex viscoelastic stress relaxation. Results indicate that analytical predictions show good agreement with CFD data (error < 5%) strictly within the core zone of high-aspect-ratio dies. However, due to the infinite-slit assumption, 3D flow kinematics near die edges induce velocity decay, leading to local deviations that require future empirical corrections. Although comprehensive physical extrusion experiments and non-isothermal viscoelastic coupling are required for industrial deployment, this semi-empirical kinematic mapping provides a foundational mathematical basis that could potentially inform future inverse die-profile design and shape distortion compensation.

## 1. Introduction

Polymer extrusion is a fundamental manufacturing process for polymeric products. Dimensional accuracy and geometric stability of the extrudate are critically influenced by extrudate swell (the Barus effect) upon exiting the die [1,2]. This phenomenon results from the release of viscoelastic stresses accumulated in the melt during flow through the confined die channel. For axisymmetric flows (e.g., through circular dies), extrudate swell is relatively uniform, and well-established predictive models exist [2,3]. In contrast, for profile extrusion through slit dies, the swell exhibits pronounced anisotropy, with systematic directional variations in the swell ratio [4,5]. However, in profile extrusion—particularly through slit dies—the swell manifests pronounced anisotropy, characterized by systematic variations in the swell ratio along different directions [6,7,8]. This non-uniform swelling critically distorts the cross-sectional profile of extrudates, posing a fundamental challenge for precision die design and product quality control [9,10,11,12]. Consequently, elucidating physical origins of anisotropic swell and establishing accurate predictive frameworks remain persistent and industrially significant scientific pursuits in polymer processing.

The phenomenon of polymer extrudate swell significantly influences extrusion die design [13,14,15,16]. Significant scholarly attention has focused on three core aspects: (1) theoretical prediction of extrudate swell ratios, (2) techniques for tracking and capturing free surface evolution during swelling [17,18], and (3) characterization of non-uniform swell behaviour. Tanner [1] established foundational formulas quantifying swell ratios for both circular and slit die geometries. Pearson et al. [5] provided comparative validation of Tanner’s formulations, extending their application to long- and short-slit dies to derive generalized computational frameworks. Tang et al. [6,7] established a tripartite methodology—encompassing rheological theory, three-dimensional viscoelastic flow simulations, and digital holography experiments—to comprehensively characterize swelling mechanisms in slit die extrudates. Researchers have conducted complementary theoretical and experimental investigations. These collective advances establish an effective methodology for swell prediction in axisymmetric configurations. Nevertheless, theoretical determination of extrudate swelling remains challenging for geometrically complex dies, necessitating the adoption of numerical simulations as the prevailing predictive approach.

The underlying mechanisms governing anisotropic extrudate swell in viscoelastic polymer flows remain incompletely characterized. While extensive studies employing theoretical frameworks, experimental techniques, and computational simulations have addressed the general phenomenon of extrudate swell, the specific origins of its non-uniform manifestations remain elusive to rigorous theoretical elucidation. This knowledge gap persists despite the established significance of anisotropic swelling in the fidelity of extrusion die design and dimensional accuracy of the product. Consequently, a systematic investigation is warranted to: (1) establish quantitative descriptors for anisotropic swell phenomena across slit dies; (2) develop predictive models for slit-flow specific anisotropy; and (3) explore the geometric and kinematic mapping rules that link local flow fields to directional swell heterogeneity.

While classical theories, notably Tanner’s elastic recovery framework [1,5], have provided foundational predictions for axisymmetric extrudate swell, they fundamentally attribute swelling to the release of stored elastic energy upon stress cessation—a mechanism implicitly assuming isotropic material response and stress-equilibrated flow at the die exit. This perspective, though successful for capillary geometries, encounters significant limitations in explaining anisotropic swelling in slit dies, where the swelling ratio varies by over 30% in different directions [6]. The long-standing dichotomy between elastic recovery and viscous redistribution mechanisms remains unresolved in such confined, non-axisymmetric flows. Proponents of the elastic recovery paradigm argue that normal-stress-driven recoil dominates swelling [1,19], whereas advocates of viscous redistribution emphasize the role of streamline reorientation and shear-induced velocity rearrangement at the free surface [7,20]. While Tanner’s stress-release approach provides a macro-scale thermodynamic perspective, the present velocity-centre hypothesis complements this by focusing on localized flow kinematics. It posits that the macroscopic anisotropic swelling profile can be mathematically correlated with multifocal reorganization of the velocity gradient field immediately downstream of the die lip. This hypothesis aims to link the spatial distribution of velocity cores within the die land to directional swell heterogeneity, thereby isolating the kinematic mapping component from complex coupled viscoelastic recovery.

To address this, the present investigation develops a theoretical framework predicated upon a multi-velocity-centre hypothesis to mathematically describe flow rearrangement processes responsible for non-uniform swelling phenomena. While the anisotropic extrudate swell of real polymers is fundamentally driven by physical viscoelastic recovery, the primary objective of this theoretical study is to explicitly isolate and map purely geometric and kinematic components of this complex phenomenon. Therefore, this paper is intended as a semi-empirical mathematical proof-of-concept for the multi-velocity-centre hypothesis.

## 2. Extrudate Swell Behaviour

Upon exiting the extrusion die, polymer melt exhibits extrudate swell (the Barus effect). For products with axisymmetric cross-sections (e.g., from circular dies), the flow is essentially one-dimensional, as the velocity profile depends only on radial position. Consequently, the resulting extrudate swell is usually uniform and isotropic. However, for profile extrusion products with non-circular cross-sections (e.g., from slit or complex dies), the swell becomes significantly anisotropic, meaning the degree of swelling varies with direction [1,7]. This anisotropy stems from several factors: (1) geometric irregularities in the flow channel, (2) distortions in the flow field caused by complex cross-sectional geometries, and (3) material nonlinearities such as viscoelastic memory effects. Furthermore, processing parameters like flow rate and temperature gradients can exacerbate anisotropic swelling.

Extrudate swell is quantitatively characterized by the extrudate swell ratio (*B*), defined as:

(1) $$B=\frac{S_{e}}{S}$$

where *S*_e_ is the stabilized cross-sectional area of extrudate in the free-swelling zone, and *S* represents the geometric cross-sectional area at the die exit.

For circular die extrusion systems, extrudate swell is alternatively quantified using the diameter swell ratio ($B_{d}$), defined as:

(2) $$B_{d}=\frac{D_{e}}{D}$$

where *D*_e_ is the stabilized diameter measured at the equilibrium swell state, and *D* denotes the nominal geometric diameter at the die exit plane.

For axisymmetric circular dies, the extrudate swell ratio (*B*) reduces to a function of diameter swell ratio ($B_{d}$):

(3) $$B=\frac{S_{e}}{S}=\frac{\pi D_{e}^{2}/4}{\pi D^{2}/4}={\left(\frac{D_{e}}{D}\right)}^{2}={\left(B_{d}\right)}^{2}$$

Building upon Tanner’s elastic recovery theory for circular extrudate swell [1], Kauzlarich [21] derived a low-shear-stress approximation for the relative extrudate swell ratio $S_{d}$:

(4) $$S_{d}\equiv B_{d}-1\approx \frac{\alpha^{2}}{12}\tau_{w}^{2}$$

where α is the Elastic parameter ($\alpha =N_{1}/\tau_{\omega }^{2}$ [s^2^/Pa], material constant), and $\tau_{\omega }$ is wall shear stress (Pa).

For fully developed laminar flow of a power-law fluid in a circular tube, the constitutive relationship between wall shear stress ($\tau_{\omega }$) and wall shear rate (${\dot{\gamma }}_{\omega }$) is given by:

(5) $$\tau_{\omega }=K{\dot{\gamma }}_{\omega }^{n}$$

where *K* is the consistency index, zero-shear viscosity parameter, and *n* is the flow behaviour index (dimensionless).

Relative extrudate swell ratio $S_{d}$ defined in Equation (4) is derived from Equation (5) through viscoelastic energy recovery theory:

(6) $$S_{d}\equiv \frac{\alpha^{2}}{12}\tau_{w}^{2}=\frac{\alpha^{2}K^{2}}{12}{\dot{\gamma }}_{w}^{2n}$$

For steady laminar flow of an incompressible power-law fluid in a circular channel, the dimensionless velocity distribution is given by [9]:

(7) $$\frac{\nu }{\nu_{\max}}=1-{\left(\frac{r}{R}\right)}^{\frac{n+1}{n}}$$

where ν is local velocity at radial position *r*, $\nu_{\max}$ is maximum velocity at the centreline, and *R* is the tube radius.

Wall shear rate (${\dot{\gamma }}_{\omega }$) for steady laminar flow of an incompressible power-law fluid is derived from the velocity gradient at the wall:

(8) $${\dot{\gamma }}_{w}=-{\left(\frac{d\nu }{dr}\right)}_{r=R}=\frac{n+1}{n}\frac{\nu_{\max}}{R}$$

The relative extrudate swell ratio ($S_{d}$) quantifying elastic recovery energy is derived from fundamental rheological parameters:

(9) $$S_{d}\equiv \frac{\alpha^{2}K^{2}}{12}{\dot{\gamma }}_{w}^{2n}=\frac{\alpha^{2}K^{2}}{12}{\left(\frac{n+1}{n}\frac{\nu_{\max}}{R}\right)}^{2n}$$

The relative extrudate swell ratio ($S_{d}$) can be expressed through the material velocity constant χ and geometric scaling:

(10) $$S_{d}\equiv \frac{\chi }{R^{2n}}\;\mathrm{where}\;\chi =\frac{\alpha^{2}K^{2}}{12}{\left(\frac{n+1}{n}\nu_{\max}\right)}^{2n}$$

where χ is the material velocity constant (Pa^2^·s^2*n*^) which combines elastic recovery (α), viscous dissipation (K), kinetic energy, and power-law index (*n*).

To bridge fundamental rheological parameters with the geometric scaling of extrudate swell, a lumped material flow constant χ is introduced. This constant links the viscoelastic memory of the polymer melt—quantified by the elastic parameter α—with its steady-state viscous dissipation, characterized by the power-law consistency index K and the shear-thinning exponent n. Furthermore, χ incorporates the kinetic energy of the flow via maximum velocity, thereby coupling the intrinsic material response with specific processing conditions. Physically, χ represents the recoverable viscoelastic energy density per unit flow history that drives the swelling upon stress cessation. Its derivation is based on low-shear-stress approximation of elastic recovery theory [1] and the fully developed velocity profile of a power-law fluid. Consequently, χ serves as a scaling parameter that combines the combined effects of fluid elasticity, shear-dependent viscosity, and flow kinematics into a single constant, enabling the subsequent geometric scaling of the relative swell ratio $S_{d}$ with the characteristic flow dimension R.

Note that the parameter χ is not a fundamental material property that can be derived solely from standard rheometry. Instead, it serves as a semi-empirical, lumped calibration parameter that implicitly captures coupling between material flow behaviour and local geometry. In practice, χ must be implicitly fitted using a baseline numerical simulation or a single-point experimental measurement.

Equation (10) provides a key scaling relationship for the relative extrudate swell ratio ($S_{d}$) under controlled flow conditions. For axisymmetric flow with constant maximum velocity ($\nu_{\max}$) and low wall shear stress ($\tau_{\omega }$), relative extrudate swell ratio $S_{d}$ is inversely proportional to $R^{2n}$ [22].

## 3. Non-Uniform Extrudate Swell Behaviour

The derivation of the multi-velocity-centre hypothesis is based on the following governing assumptions to ensure scientific transparency and mathematical reproducibility:

(1) The fluid is considered incompressible, ensuring volumetric flow conservation.
(2) The flow is fully developed within the slit die and isothermal conditions are maintained.
(3) The local swelling contribution from any discrete velocity element decays radially according to an inverse square distance relationship.

**Figure 1:**
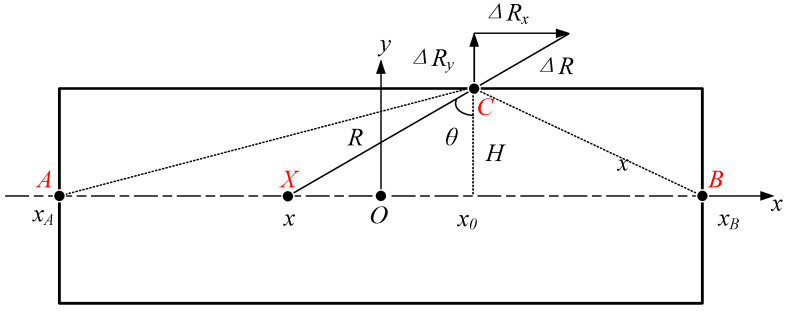
Non-uniform behaviour of extrudate swell in slit extrusion.

For slit-extruded profiles, both experiments and simulations reveal pronounced anisotropy [7,8], where the swell ratio differs significantly depending on the measurement direction (Figure 1). Unlike circular dies which exhibit point-symmetric flow with a singular velocity maximum at the centreline, slit dies (aspect ratio > 10) develop a planar-symmetric flow. This symmetry, defined by the plane (y=0) where shear stress $\tau_{\omega }=0$, leads to anisotropic swelling through differential stress recovery, expressed as:

(11) $$B_{w}\neq B_{t,edge}\neq B_{t,middle}$$

where $B_{w}$ and $B_{t}$ are the width and thickness swell ratio, respectively.

To investigate this correlation, a theoretical model for non-uniform behaviour of extrudate swell in slit extrusion is developed, as shown in Figure 1. The X coordinates of points A and B are 0 mm and 20 mm, respectively. The extrudate swell of slit extrusion products is symmetrical about the axis, so half of it can be studied. Leveraging the symmetry condition (${\left.{\nabla \tau }_{xy}\right|}_{y=0}=0$), a computationally efficient half-domain modelling is employed.

Figure 1 illustrates the theoretical model. Line segment AB represents the symmetry plane (y = 0) of the fluid flow within the straight slit die channel. Point *X* denotes an arbitrary position along this symmetry plane, while point C is located on the die wall surface. The channel height is defined as 2H, exhibiting symmetric geometry about the symmetry plane. As illustrated, the radius *R* here is the distance between points X and C, which follows the geometric relationship:

(12) $$R=\frac{H}{\cos\theta }$$

where *H* is the half-channel height, and θ is the angle between line *XC* and the slit channel’s vertical axis.

At any arbitrary point *X* along the symmetry plane (y = 0), the diameter swell ratio $B_{d}$ at point C on the extrudate surface is defined by the geometric deformation following extrusion:

(13) $$B_{d}=\frac{R_{e}}{R}$$

where *R*_e_ is the distance *R* after elastic recovery in the extrudate.

Derived from Equation (13), the radial displacement vector magnitude ΔR resulting from extrusion swell at material point C is expressed as:

(14) $$\Delta R=B_{d}R-R=\left(B_{d}-1\right)\frac{H}{\cos\theta }$$

The swell-induced displacement of material point C is resolved into orthogonal components relative to the die coordinate system:

(15) $$\Delta R_{x}=\Delta R\sin\theta =\left(B_{d}-1\right)H\tan\theta$$

(16) $$\Delta R_{y}=\Delta R\cos\theta =\left(B_{d}-1\right)H$$

where $\Delta R_{x}$ is lateral displacement along the die width dimension (*X*-axis), and $\Delta R_{y}$ is the thickness-direction displacement (*Y*-axis).

Equation (17) resolves lateral displacement through geometric parametrization of Figure 1:

(17) $$\Delta R_{x}=\left(B_{d}-1\right)H\tan\theta =\left(B_{d}-1\right)\left(x_{0}-x\right)$$

where $x_{0}$ is the normal projection coordinate of point C on the die centreline (*X*-axis), and x identifies the velocity centreline position of material point X.

The derivation follows the kinematic relationship $\tan\theta =\frac{\left|x-x_{0}\right|}{H}$ per Figure 1, confirming a linear dependence on coordinate separation.

Displacement components $\Delta R_{x}$ and $\Delta R_{y}$ arise from cumulative contributions of all velocity elements along the centreline. For any material point *X* on this centreline (spanning $\left[x_{A},x_{B}\right]$), total displacements at observation point *C* are obtained through integration over the entire domain:

(18) $$\Delta R_{x}^{T}=\int_{x_{A}}^{x_{B}}\Delta R_{x}dx=\int_{x_{A}}^{x_{B}}\left(B_{d}-1\right)\left(x_{0}-x\right)dx$$

(19) $$\Delta R_{y}^{T}=\int_{x_{A}}^{x_{B}}\Delta R_{y}dx=\int_{x_{A}}^{x_{B}}\left(B_{d}-1\right)Hdx$$

where $\Delta R_{x}^{T}$, $\Delta R_{y}^{T}$: total displacement vectors in the width (*X*) and thickness (*Y*) directions at *C*; $x_{A}$, $x_{B}$: boundary coordinates defining the velocity centreline segment (Figure 1); integral operators: integrate the localized kinematic swelling contributions along the symmetry plane.

From Equation (10), defining the radial swell ratio, the displacement integrals in the *x*- and *y*-directions are derived as follows:

(20) $$\begin{array}{l} \Delta R_{x}^{T}=\int_{x_{A}}^{x_{B}}\left(B_{d}-1\right)\left(x_{0}-x\right)dx\\ =\int_{x_{A}}^{x_{B}}S_{d}\left(x_{0}-x\right)dx\\ =\int_{x_{A}}^{x_{B}}\frac{\chi }{R^{2n}}\left(x_{0}-x\right)dx \end{array}$$

(21) $$\begin{array}{l} \Delta R_{y}^{T}=\int_{x_{A}}^{x_{B}}\left(B_{d}-1\right)Hdx\\ =\int_{x_{A}}^{x_{B}}S_{d}Hdx=\int_{x_{A}}^{x_{B}}\frac{\chi }{R^{2n}}Hdx \end{array}$$

Total displacement in the *x*- and *y*-directions due to non-uniform extrudate swell is quantified by the integrals:

(22) $$\Delta R_{x}^{T}=\chi \int_{x_{A}}^{x_{B}}\frac{\left(x_{0}-x\right)}{R^{2n}}dx=\chi f_{x}\left(x\right)$$

(23) $$\Delta R_{y}^{T}=\chi \int_{x_{A}}^{x_{B}}\frac{H}{R^{2n}}dx=\chi f_{y}(x)$$

where χ is the semi-empirical calibration parameter, $f_{x}\left(x\right)$ and $f_{y}\left(x\right)$ represent the geometric integral functions in the *x*- and *y*-directions, respectively.

(24) $$f_{x}\left(x\right)=\int_{x_{A}}^{x_{B}}\frac{x_{0}-x}{R^{2n}}dx$$

(25) $$f_{y}(x)=\int_{x_{A}}^{x_{B}}\frac{H}{R^{2n}}dx$$

To prevent integration ambiguity, let $x_{0}$ denote the fixed geometric evaluation point on the cross-section, and let x act strictly as the dummy integration variable along the die width. Distance R (as depicted in Figure 1) between the flow element and the evaluation point is consistently defined as

(26) $$R=\sqrt{{\left(x_{0}-x\right)}^{2}+H^{2}}$$

For non-Newtonian fluids, the power-law index (*n*) ranges from 0 to 1. The total swell ratio in the *x*-direction at point *C* is derived from the integral expression governing swell behaviour:

(27) $$\begin{array}{l} f_{x}\left(x\right)=\int_{x_{A}}^{x_{B}}\frac{x_{0}-x}{R^{2n}}dx=\int_{x_{A}}^{x_{B}}\frac{x_{0}-x}{{\left({\left(x_{0}-x\right)}^{2}+H^{2}\right)}^{n}}dx\\ =-\frac{{\left({\left(x_{0}-x\right)}^{2}+H^{2}\right)}^{1-n}}{2-2n}\left|\begin{array}{c} x_{B}\\ x_{A} \end{array}\right. \end{array}$$

For power-law fluids with an index of *n* = 0.5, the total swell ratio in the *x*-direction at any coordinate $x_{0}$ on the extrudate profile due to anisotropic extrudate swell is defined by integral expression:

(28) $$f_{x}\left(x_{0}\right)=\sqrt{{\left(x_{0}-x_{A}\right)}^{2}+H^{2}}-\sqrt{{\left(x_{0}-x_{B}\right)}^{2}+H^{2}}$$

**Figure 2:**
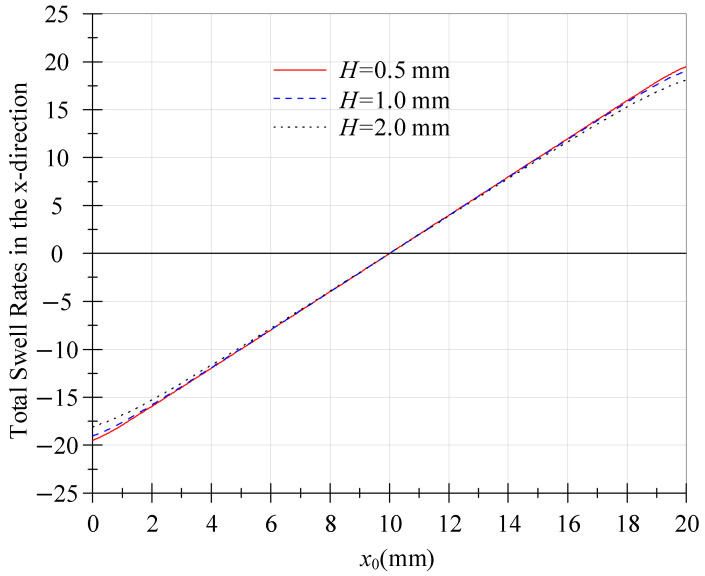
Total swell rates in the *x*-direction.

To characterize the geometric dependence of anisotropic extrudate swell, slit channel half-heights (*H*) of 0.5, 1.0, and 2.0 mm were simulated with a fixed width (*W*) of 20 mm and a power-law index *n* = 0.5. Equation (28) governs the predicted *x*-direction swell ratios. As shown in Figure 2, swell ratios scale with H due to its direct proportionality to $f_{x}\left(x_{0}\right)$, while the distribution pattern remains height-invariant. Notably, the displacement profile exhibits antisymmetric behaviour about the slit centreline ($x_{0}=10$).

The total swell ratio in the *y*-direction at point *C* is derived from the integral expression governing swell behaviour:

(29) $$f_{y}(x)=\int_{x_{A}}^{x_{B}}\frac{H}{{\left({\left(x_{0}-x\right)}^{2}+H^{2}\right)}^{n}}dx$$

For power-law fluids with an index of *n* = 0.5, the total swell ratio in the *y*-direction at any coordinate $x_{0}$ on the extrudate profile due to anisotropic extrudate swell is defined by integral expression:

(30) $$\begin{array}{l} f_{y}(x_{0})=\int_{x_{A}}^{x_{B}}\frac{H}{\sqrt{{\left(x_{0}-x\right)}^{2}+H^{2}}}dx\\ =H\mathrm{Arcsinh}\left(\frac{x-x_{0}}{\left|H\right|}\right)\left|\begin{array}{c} x_{B}\\ x_{A} \end{array}\right.\\ =H\ln\frac{\left(x_{B}-x_{0}\right)+\sqrt{{\left(x_{B}-x_{0}\right)}^{2}+H^{2}}}{\left(x_{A}-x_{0}\right)+\sqrt{{\left(x_{A}-x_{0}\right)}^{2}+H^{2}}} \end{array}$$

Function $f_{y}(x_{0})$ attains its maximum at the slit centreline, $x_{0}=(x_{A}+x_{B})/2$, where its first derivative is zero. At this critical coordinate, $f_{y}\left(x_{0}\right)$ attains its global maximum value, corresponding to the peak swelling displacement at the slit die centreline. Functional behaviour exhibits strict monotonicity.

**Figure 3:**
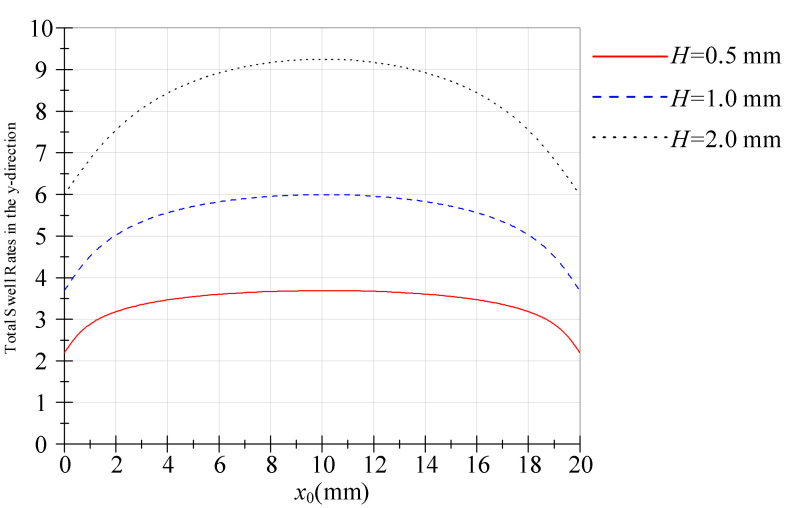
Total swell rates in the *y*-direction.

The transverse strain evolution along the streamwise coordinate is illustrated in Figure 3. As derived from Equation (30), the *y*-direction swell profile exhibits characteristic parabolic symmetry with a maximum at *x*_0_ = 10 mm, indicating strain localization at the flow centreline. The reduction in expansion toward the edges (*x*_0_ = 0 and *x*_0_ = 20) is a direct consequence of 3D edge effects and the presence of side-wall constraints. Unlike the central region which approximates 2D planar Poiseuille flow, the corners are subjected to multi-axial shear that modifies local velocity gradients and restricts purely kinematic rearrangement.

The resultant extrudate swelling emerges from coupled swell effects along orthogonal coordinates. Continuum mechanics analysis of slot extrusion phenomena reveals a fundamental mapping relationship between die geometry coordinates $r={\left[x,y\right]}^{T}$ and extrudate profiles $r_{e}=\left[x_{e},y_{e}\right]$:

(31) $$\left[\begin{array}{c} x_{E}\\ y_{E} \end{array}\right]=\left[\begin{array}{cc} x & 1\\ y & 1 \end{array}\right]A=\left[\begin{array}{cc} x & 1\\ y & 1 \end{array}\right]\left[\begin{array}{cc} 1 & 1\\ \Delta R_{x}^{T} & \Delta R_{y}^{T} \end{array}\right]$$

where $\Delta R_{x}^{T}$ and $\Delta R_{y}^{T}$ denote total displacement tensors and A represents the strain transformation operator. The invertibility of A establishes:

(i) A predictive framework for anisotropic swelling via forward strain-field computation.
(ii) An inverse rheological design methodology for die lip contour synthesis.

## 4. Numerical Verification

**Figure 4:**
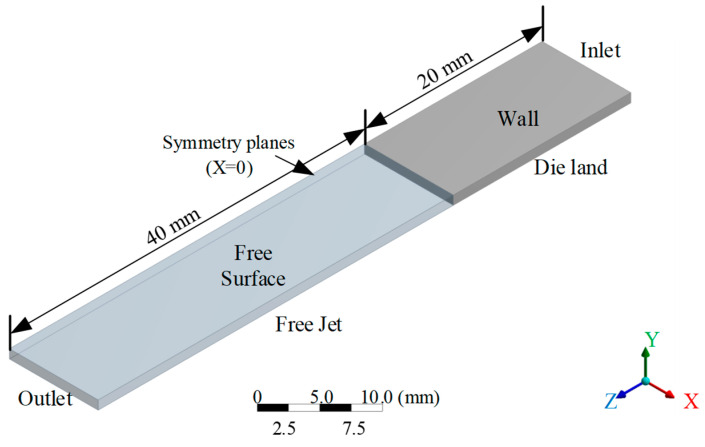
Computational domain of slit die.

In this study, the simulated slit die features a specific aspect ratio of 10. Three-dimensional isothermal flow simulations of power-law fluids (K = 1000 Pa·s, *n* = 0.5) through confined slit dies were conducted using the ANSYS Polyflow 2022 R1 finite-element framework. These isothermal simulations employing an inelastic power-law model were designed to isolate geometric and kinematic aspects of the multi-velocity-centre hypothesis, thereby circumventing the complexities of viscoelastic stress relaxation and thermal effects inherent to real polymer melts. The computational domain (Figure 4) comprised a 20 mm die land (L) and 40 mm free-jet section ($L_{F}$). Slit dimensions were a gap height (2*H*) of 2 mm and a width (*W*) of 20 mm, giving an aspect ratio (*W*/2*H*) of 10. Leveraging geometric symmetry, the model employed a quarter-domain formulation to optimize computational efficiency. The spatial discretization employed graded elements across key dimensions: the height direction used 10 elements (bias factor 4), the width direction used 40 elements with end refinement (bias 4), the die land was discretized with 30 axial elements refined at the inlet and outlet (bias 6), and the free-jet section used 35 elements with clustering near the exit zone (bias 13).

A mesh sensitivity analysis was performed to ensure grid-independent results. The baseline mesh was iteratively refined and coarsened, varying the global node count by approximately ±5%. Mesh convergence was deemed achieved when variation in the primary extrudate swell ratios between successive mesh generations was less than 1%. The mesh satisfying this criterion was adopted for all subsequent simulations. The final mesh selected for all production runs satisfied this criterion. For the steady-state solver, a stringent convergence criterion required that the normalized residuals for mass, momentum, and constitutive equations all fall below a threshold of 10^−6^, ensuring sufficient solution accuracy for the nonlinear flow fields. These measures collectively confirm that the presented simulation results are quantitatively robust and independent of numerical discretization parameters.

Boundary conditions were applied as follows:

(1) Inlet ($\Gamma_{\mathrm{in}}$): A fully developed flow profile with a volumetric flow rate of *Q* = 200 mm^3^/s.
(2) Walls ($\Gamma_{\mathrm{wall}}$): No-slip condition (v=0).
(3) Outlet ($\Gamma_{\mathrm{out}}$): Stress-free boundary (σ⋅n=0).
(4) Free surfaces ($\Gamma_{\mathrm{free}}$): Kinematic condition (v⋅n=0) with zero traction force.
(5) Symmetry planes ($\Gamma_{\mathrm{sym}}$): Mirror symmetry (v⋅n=0, t⋅σ⋅n=0).

**Figure 5:**
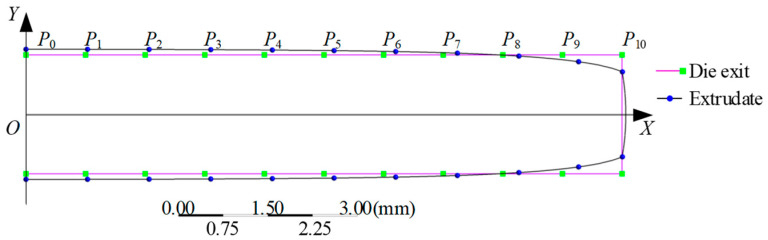
Profile comparison between die exit and extrudate.

Analysis of the numerical results (Figure 5) reveals that geometric symmetry about the *x*-axis permits evaluation of half-domain extrusion behaviour; along this coordinate, the *x*-direction swelling displacement—defined as the spatial offset between die exit and extrudate profiles—displays a monotonic increase aligned with the predictive curve, while *y*-direction displacement fields exhibit parabolic spatial progression consistent with theoretical distribution, confirming non-uniform extrudate swell kinematics.

**Figure 6:**
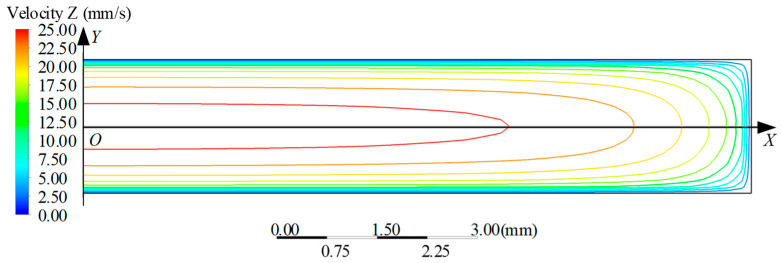
Velocity distribution at die exit.

Velocity distribution at the slit die exit is illustrated in Figure 6, revealing that while a velocity core along the *x*-axis exhibits uniform magnitude (with isovels parallel to this axis) at central regions—consistent with infinitely broad slit assumptions for 1D flow—lateral decay initiates proximal to |*x*| = 6 mm, manifesting as curved isovels indicative of transitional 2D flow behaviour. Consequently, quantitative comparison between theoretical frameworks (relying on multi-centre velocity homogeneity) and numerical simulations is confined to the symmetry-preserving domain *x* ∈ [−6, 6] mm, where centreline velocity invariance remains experimentally validated.

A comparison of extrudate swell between numerical simulations and theoretical predictions is shown in Table 1. Quantitative agreement is achieved between numerical simulations and theoretical predictions at χ=0.016.

**Figure 7:**
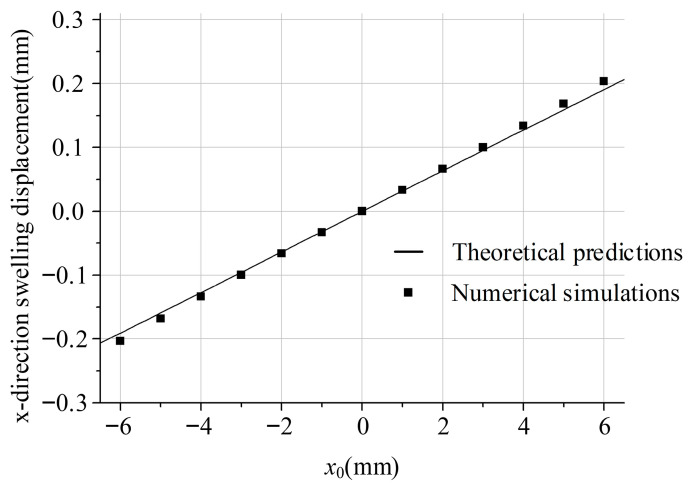
*x*-direction swelling displacement (*H* = 1 mm).

As shown in Figure 7, the *x*-direction swelling displacement profiles from both the theoretical model and numerical simulations exhibit a consistent progression. However, simulated values (see Table 1 for details) show a slightly steeper gradient than the theoretical curve. This discrepancy indicates amplified edge effects in the near-wall regions, likely due to localized viscous dissipation and velocity decay near the boundaries, phenomena not fully captured by the theoretical velocity core assumption.

**Figure 8:**
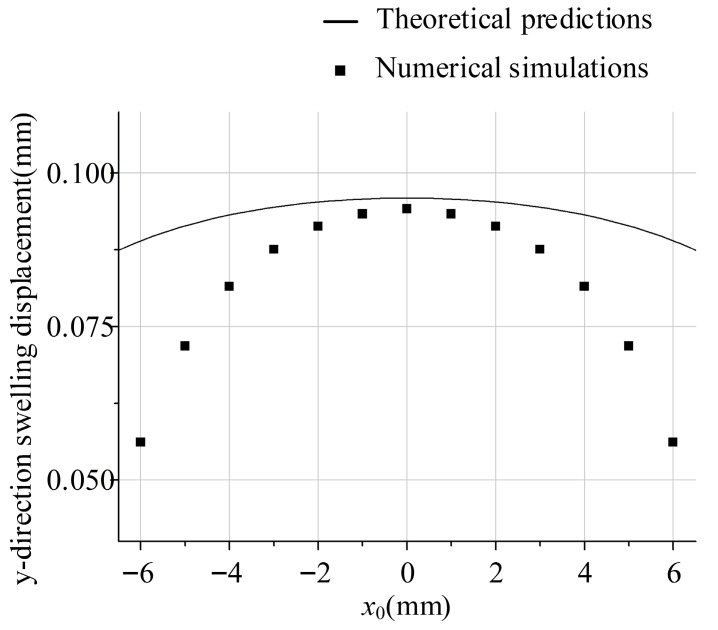
*y*-direction swelling displacement (*H* = 1 mm).

Figure 8 reveals systemic alignment between theoretical and simulated *y*-direction swelling displacement profiles along the *x*-axis (Table 1), yet divergent behaviour emerges in boundary regions (|*x*|→6 mm). This divergence primarily originates from the infinite-slit assumption in theoretical derivation, wherein uniform velocity along the centreline (∂v/∂x≈0) contrasts with experimental velocity decay. Consequently, while *x*-directional displacement—dominated by far-field inertial effects—exhibits <5% deviation, *y*-directional displacement shows greater variance due to localized viscous dissipation in low-velocity boundary layers, consistent with non-Newtonian stress relaxation characteristics at *n* = 0.5.

The present theoretical model is grounded in the “infinitely wide slit” assumption, which postulates a uniform velocity profile in the width direction (i.e., ∂v/∂x ≡ 0). This simplification reduces the flow to a one-dimensional problem, enabling an analytical formulation consistent with classical die-swell theories that attribute swelling primarily to elastic recovery upon stress release [1,5]. However, as evidenced by numerical simulations (Figure 7), a discernible velocity decay emerges in the boundary regions (|x|→6 mm), signifying a transition from idealized one-dimensional flow to a more complex two-dimensional behaviour near the edges. This deviation directly underpins the observed discrepancies in the *y*-direction swelling displacement (Figure 8), where theoretical predictions diverge from simulated values at larger |*x*|. The velocity decay in these regions intensifies localized viscous dissipation and alters the stress relaxation dynamics—effects that are inherently omitted in the infinite-slit framework, particularly for shear-thinning power-law fluids (e.g., *n* = 0.5) [6]. As demonstrated, the high-accuracy agreement (errors < 5%) is firmly restricted to the core zone (|x|<3 mm). Beyond this region, the infinite-slit assumption breaks down due to significant velocity decay near the side walls, causing the analytical predictions to deviate from CFD results with local edge errors exceeding 30%.

Despite this limitation, the model remains notably applicable and accurate for dies with high aspect ratios (*W/2H* > 10). In such configurations, the core flow domain (approximately |*x*| < 3 mm) retains a nearly uniform velocity, thereby upholding the key model assumption. Consequently, theoretical predictions align well with simulations in the central zone, with minimal deviation (e.g., <5% in the *x*-direction displacement). For comprehensive design applications, especially where edge precision is critical, future model enhancements could integrate edge-effect corrections derived from 3D flow analyses [7].

## 5. Scope and Limitations

The multi-velocity-centre hypothesis provides a mathematical mapping for anisotropic swell. However, it is crucial to acknowledge the fundamental limitations of the current theoretical framework and its numerical verification, which point to necessary directions for future research.

First, numerical verification is confined to an inelastic, isothermal power-law fluid (*n* = 0.5). Given that real polymer melts exhibit strong viscoelastic memory, this CFD validation primarily tests geometric and kinematic consistency of the proposed mapping, not the full physics of viscoelastic stress relaxation.

Second, the isothermal assumption neglects practical thermal effects. Temperature and viscosity gradients near the die walls can shift the velocity core and alter the anisotropic swell profile.

Third, the model’s infinite-slit assumption (∂v/∂x≈0) fails near the side walls, as evidenced by our results. Consequently, accurate predictions are confined to the core zone of high-aspect-ratio dies. For industrial dies with finite width, future work must incorporate edge-correction functions or hybrid CFD strategies.

Therefore, to transform this mathematical proof-of-concept into a predictive engineering tool, comprehensive physical extrusion experiments and non-isothermal viscoelastic simulations are essential next steps.

## 6. Conclusions

In this study, a theoretical mathematical framework is developed to geometrically describe the anisotropic extrudate swell profile of polymer melts in rectangular slit dies. Based on a detailed geometric and kinematic analysis, the following main conclusions are drawn:

(1) Proof-of-Concept for Kinematic Mapping: Recognizing that actual polymer extrudate swell is fundamentally driven by viscoelastic recovery, this study explored a multi-velocity-centre hypothesis to approximate geometric and kinematic components of this phenomenon. This approach serves as a mathematical proof-of-concept, adapting the macroscopic diameter swell ratio to estimate the localized swelling profile.
(2) Semi-Empirical Mathematical Formulation: By eliminating redundant geometric decompositions, a simplified analytical integration scheme was formulated. The model relies on the introduction of χ, which is strictly defined as a semi-empirical, lumped calibration parameter rather than a fundamental material property. This parameter serves as a proxy to capture the apparent local coupling between flow kinematics and macroscopic swelling.
(3) Core-Zone Accuracy and Edge Limitations: The framework was subjected to a numerical test for kinematic consistency using isothermal, inelastic power-law fluid (*n* = 0.5) CFD simulations. The analytical predictions show good agreement (errors < 5%) strictly within the core zone of high-aspect-ratio dies. However, the infinite-slit assumption causes the model to break down in the edge regions, where 3D velocity decay leads to localized deviations exceeding 30%.
(4) Future Prospects and Engineering Application: To transition this purely kinematic mapping into a generalized predictive model, future research must incorporate comprehensive physical extrusion experiments, non-isothermal viscoelastic constitutive modelling, and empirical edge-correction functions. Despite current limitations, this semi-empirical framework offers an initial geometric approach that may assist in the future development of inverse die-profile optimization and shape distortion compensation algorithms.

## Figures and Tables

**Table 1:** Comparison of extrudate swell profile.

x	$\Delta R_{x}^{T}$ (mm)			$\Delta R_{y}^{T}$ (mm)		
	Theoretical	Numerical	Error (%)	Theoretical	Numerical	Error (%)
0	0.000	0.000	0.000	0.096	0.094	2.083
1	0.032	0.033	3.125	0.096	0.093	3.125
2	0.064	0.066	3.125	0.095	0.091	4.211
3	0.095	0.100	5.263	0.094	0.088	6.383
4	0.127	0.134	5.512	0.093	0.081	12.903
5	0.159	0.168	5.660	0.091	0.072	20.879
6	0.191	0.203	6.283	0.089	0.056	37.079

## Data Availability

All data needed to evaluate the conclusions in the paper are present in the paper. All raw data are available from the corresponding author upon reasonable request.
